# The *in vitro* immunogenic potential of caspase-3 proficient breast cancer cells with basal low immunogenicity is increased by hypofractionated irradiation

**DOI:** 10.1186/s13014-015-0506-5

**Published:** 2015-09-17

**Authors:** Bernhard Kötter, Benjamin Frey, Markus Winderl, Yvonne Rubner, Heike Scheithauer, Renate Sieber, Rainer Fietkau, Udo S. Gaipl

**Affiliations:** Department of Radiation Oncology, University Hospital Erlangen, Friedrich-Alexander-Universität Erlangen-Nürnberg, Erlangen, Germany; Department of Radiotherapy and Radiation Oncology, Ludwig Maximilian University Munich, D-81377 Munich, Germany

## Abstract

**Background:**

Radiotherapy is an integral part of breast cancer treatment. Immune activating properties of especially hypofractionated irradiation are in the spotlight of clinicians, besides the well-known effects of radiotherapy on cell cycle and the reduction of the clonogenic potential of tumor cells. Especially combination of radiotherapy with further immune stimulation induces immune-mediated anti-tumor responses. We therefore examined whether hypofractionated irradiation alone or in combination with hyperthermia as immune stimulants is capable of inducing breast cancer cells with immunogenic potential.

**Methods:**

Clonogenic assay, AnnexinA5-FITC/Propidium iodide assay and ELISA analyses of heat shock protein 70 and high mobility group box 1 protein were applied to characterize colony forming capability, cell death induction, cell death forms and release of danger signals by breast cancer cells in response to hypofractionated radiation (4x4Gy, 6x3Gy) alone and in combination with hyperthermia (41.5 °C for 1 h). Caspase-3 deficient, hormone receptor positive, p53 wild type MCF-7 and caspase-3 intact, hormone receptor negative, p53 mutated MDA-MB231 breast cancer cells, the latter in absence or presence of the pan-caspase inhibitor zVAD-fmk, were used. Supernatants of the treated tumor cells were analyzed for their potential to alter the surface expression of activation markers on human-monocyte-derived dendritic cells.

**Results:**

Irradiation reduced the clonogenicity of caspase deficient MCF-7 cells more than of MDA-B231 cells. In contrast, higher amounts of apoptotic and necrotic cells were induced in MDA-B231 cells after single irradiation with 4Gy, 10Gy, or 20Gy or after hypofractionated irradiation with 4x4Gy or 6x3Gy. MDA-B231 cells consecutively released higher amounts of Hsp70 and HMGB1 after hypofractionated irradiation. However, only the release of Hsp70 was further increased by hyperthermia. Both, apoptosis induction and release of the danger signals, was dependent on caspase-3. Only supernatants of MDA-B231 cells after hypofractionated irradiation resulted in slight changes of activation markers on dendritic cells; especially that of CD86 was upregulated and HT did not further impact on it.

**Conclusions:**

Hypofractionated irradiation is the main stimulus for cell death induction and consecutive dendritic cell activation in caspase proficient breast cancer cells. For the assessment of radiosensitivity and immunological effects of radio- and immunotherapies the readout system is crucial.

**Electronic supplementary material:**

The online version of this article (doi:10.1186/s13014-015-0506-5) contains supplementary material, which is available to authorized users.

## Background

With approximately 70.000 new cases of disease per year, breast cancer (mamma carcinoma) represents the most frequent and, along with approximately 17.000 deaths per year, also the deadliest cancer disease for women in Germany. One out of 8 German women will suffer from mamma carcinoma during lifetime. This implies that deep knowledge about breast cancer development, mechanisms of tumor progression and related treatments is mandatory. The main risk factors to develop a mamma carcinoma are female gender and seniority (>60 years). Breast cancer displays a heterogeneous tumor disease and multiple subtypes exist [1]. Ductal, originating from lactiferous ducts, are to be differed from lobular carcinomas, originating from glandular lobes. With about 70 % of the cases the invasive ductal carcinoma is the prominent type [2]. Precancerous conditions are the Ductal Carcinoma *in situ* (DCIS) and the Carcinoma Lobulare *in situ* (CLIS), of which the DCIS shows the more aggressive progress and in about a third to half of the cases develops to an invasive carcinoma within 10–20 years [3]. Benign and malignant pre-existing conditions of the breast, genetic mutations, most prominent in the BRCA (Breast Cancer) gene, positive family history, long period of estrogen-exposure (early menarche, late menopause, obesity) and life style are main risk factors [4].

Triple negative breast cancer (TNBC) represents 15–20 % of all breast cancers that lack estrogen receptor (ER) and progesterone receptor (PgR) expression as well as amplification of the human epidermal growth factor receptor 2 (HER2). TNBCs are an aggressive group of breast cancers with higher rates of relapse and to date not a single targeted therapy has been approved for its treatment [5]. Combinational effects of chemotherapy, photothermal therapy, and gene therapy with low drug dose are currently tested as promising strategy for TNBC treatment [6]. However, a relative radioresistance for TNBC does not imply radiation omission, because radiotherapy (RT) provides an absolute loco-regional risk reduction [7].

RT is therefore a crucial component for the treatment of breast cancer [8]. Commonly it is applied in daily fractions of 1.8–2 Gy up to a total dose of 50 Gy [9]. However, long term follow-up confirms that appropriately dosed hypofractionated radiotherapy is safe and effective for patients with early breast cancer [10]. Meanwhile, the use of fractions >2.0 Gy (hypofractionation) is standard in the UK, and increasingly used internationally for this tumor entity [11]. The results of the German multicenter phase II trial (ARO-2010-01) also suggest that hypofractionation with simultaneous integrated boost for early breast cancer is feasible [12]. However, integration of RT in multimodal breast cancer treatment still remains a challenge [13].

Emerging evidence suggests that besides inducing local DNA damage, RT promotes a pro-immunogenic milieu within the tumor capable of stimulating host cancer-specific immune responses. Immunogenic breast cancer cell death stimulated by either high-dose RT alone or concurrent chemoradiation regimens may contribute to this [14]. Especially combination of RT with further immune stimulation contributes to immune-mediated abscopal effects of RT [15, 16]. Recent *in vitro* and *in vivo* studies indicate a unique immune modulating prospect of hyperthermia (HT), especially when combined with RT [17].

Regional or local HT describes the overheating of certain body parts by using electromagnetic radiation, e.g. microwaves. Temperatures of 40–44 °C for a period of at least 60 min should be achieved for therapeutic effectiveness [18]. Of note is that recommendations for the implementation of quality-assured hyperthermia treatments should be followed when applying HT in multimodal therapy settings [19]. A radiosensitizing effect of HT for the treatment of breast cancer has been discussed for long time [20, 21]. In the update of 2012 of the AGO German interdisciplinary S3-guidelines for diagnostic, therapy and follow-up care of breast cancer it is stated that for chest wall recurrences, simultaneous chemotherapy or hyperthermia as radiosensitizing methods may result in higher response rates. For re-irradiation, combination of RT with HT received the Oxford Levels of Evidence of 1b in these guidelines. A Meta-analysis of 23 studies with 1861 patients with tumors of the chest wall, cervix, rectum, bladder, prostate, head-neck-region and melanoma points out a highly significant benefit regarding loco-regional tumor control when RT is combined with HT [22].

Oldenborg and colleagues proposed and examined a hypofractionated irradiation with a single dose of 4 Gy or 3 Gy, respectively, twice a week plus HT for patients with recurrent breast cancer in previously irradiated area [23]. Of note is that no increasing toxicity was observed when adding HT to RT for the treatment of high risk breast carcinoma [24]. Re-irradiation in combination with HT is an effective and a safe modality to treat loco-regional recurrences of breast cancers [25]. Besides HT, also electrochemotherapy is discussed to be beneficial for chest wall breast cancer recurrence [26].

The dogma of classical radiobiology states that the cytotoxic effects of irradiation on tumor cells are due to induction of DNA double strand breaks. The colony forming assay is used as gold standard to determine the “viability” of cells as defined by bearing the capability to form colonies >50 cells. However, no predictions about cell death of a single cell and forms of tumor cell death including their immunogenicity can be drawn by this assay [27], besides several other draw backs such as seeding the cells from the beginning in different concentrations in dependence of the treatment, disregarded cell-to-cell communication, clump artifacts and unification of colonies of different sizes [28].

None repaired DNA damage can lead to cell death via apoptosis, mitotic catastrophe, autophagy, or terminal growth arrest senescence [29]. Defining life and death is more problematic than one would guess and regulated cell death often results in initiation of secondary regulated cell death accompanied by inflammatory events [30]. The success of RT does therefore not only derive from direct cytotoxic effects on the tumor cells alone, but instead might also depend on resulting innate as well as adaptive immune responses [31]. The latter can be initiated by immunogenic cell death forms such as necrosis (summarised in [32]).

While apoptotic cells when present in physiological amounts exert non- or even anti-inflammatory effects [33], primary and secondary necrotic cells lead to an activation of the immune response by release of danger signals such as high mobility group box protein 1 (HMGB1), Adenosine-Triphosphate (ATP) and/or heat shock protein 70 (Hsp70) (summarised in [34]). Secondary necrotic cells derive from apoptotic ones that have lost their membrane integrity during the course of cell death execution [35]. Therefore they are also often termed as late apoptotic cells. However, following the definition of necrosis, namely the loss of membrane integrity, secondary necrosis is the better fitting term from the immunological point of view.

Central in immune activation against the tumor by therapy-induced immunogenic tumor cell death forms are dendritic cells (DCs). High numbers of apoptotic cells and danger signals released by primary and secondary necrotic cells trigger maturation and activation of DCs [36, 37]. The latter then present tumor-derived antigen to CD8+ T cells and thereby initiate cytotoxic T cell responses against the tumor (summarised in [38]).

Hence, based on the clinical launching of hypofractionated RT and addition of HT as radiosensitizer for the treatment of breast cancer, our study opened the question whether cell death forms and immunogenic potential of breast cancer cells differs after hypofractionated RT and/or HT. We focused on the MDA-MB231 breast cancer cell line representing triple-negative mesenchymal highly invasive human breast cancer cells and MCF-7 breast cancer cells, being positive for ER and PgR expression, but deficient for caspase-3, for comparison. As activation of caspases is widely considered as the initiator mechanism of regulated cell death [30], we especially focused on the role caspase-3 in induction of cell death with immunogenic potential of breast cancer cells.

## Materials and methods

### Cell culture

MCF-7 (ER positive; PgR positive; p53 wt; caspase-3 deficient [39, 40]) representing luminal-epithelial weakly invasive [41] and MDA-MB231 (ER negative; PgR negative; p53 mutated; caspase-3 intact) [42] representing triple-negative mesenchymal highly invasive [41] human breast cancer cells were grown in Dulbecco’s modified eagle medium (DMEM, PAN-Biotech, Germany) at 37 °C, 5 % CO_2_ and 90 % humidity to 70-80 % confluence. 10 % fetal bovine serum (FBS superior, Biochrom, Germany) and 0,4 μl/ml Gentamicin were added to optimize the growing situation. Both cell lines were obtained from the American Type Culture Collection (ATCC, Germany) and tested to be free of mycoplasma contamination.

### Treatment of the tumor cells with ionizing irradiation and/or hyperthermia

Irradiation of the cells was performed with an X-ray generator (120 kV, 22.7 mA, variable time; GE Inspection Technologies, Hürth, Germany). A dose of 2, 4, 10 or 20Gy was applied for irradiation with a single dose and 4x4Gy or 6x3Gy for the fractionated treatments. The latter treatment schemes should mimic in our preclinical *in vitro* model system clinically relevant hypofractionated irradiation schemes as proposed by Oldenborg and colleagues: single fractions of 4Gy (8 times; two fractions a week) or of 3Gy (12 times; two fractions a week) [23]. Hyperthermia (HT) at 41.5 °C for 1 h was applied 4 h before or after irradiation, mimicking the time window of combined application of RT and HT in the clinics [43]. For HT treatment, a HT-chamber constructed by our physicists was used [44]. Fractionated irradiation was combined with HT on the first and last day of the distinct fractionation scheme. To block caspases in caspase 3 proficient MDA-MB231 cells, the pan-caspase inhibitor zVAD-fmk was added to the cell cultures in a concentration of 50 μM at day one and three or four during hypofractionated irradiation with 4x4Gy or 6x3Gy, respectively.

### Colony formation assay

The descent of proliferation competent and therefore colony forming tumor cells after the respective treatments was assessed with clonogenic assays. For this, the cells were plated in triplicates in 60-mm dishes (Nunc Thermo Fisher, Waltham, USA) at concentrations estimated to yield 40–150 colonies/dish. Afterwards, the cells were treated with a single dose irradiation of 1, 2, 4, 6, 8, or 10Gy with or without hyperthermia 4 h before or after the irradiation. After incubation for approximately two weeks, the cells were fixed with methylene blue (Sigma-Aldrich, Munich, Germany) for 30 min. Colonies with >50 cells were scored. The calculation of the respective linear quadratic fitting curves upon the colony forming assays was performed with the R package ‘CFAssay’ (version 1.2.0.) on R 3.2.1 [45].

### Analyses of cell death forms and cell cycle phases with flow cytometry

To identify different forms of tumor cell death, the exposure of phosphatidylserine on the surface of apoptotic cells was analyzed using FITC-labeled AnnexinV (AnxA5-FITC). To distinguish apoptotic cells from necrotic ones, the co-staining with propidium iodide (PI) was applied as described by Koopman et al. [46]. Apoptotic cells are referred to as being positive for AnxA5 binding and negative for PI intercalation into the DNA, and necrotic ones are positive for both, AnxA5 and PI binding. Necrotic ones can be further distinguished in primary necrotic ones with a DNA content of viable cells (PI++) and secondary necrotic/late apoptotic ones with already reduced DNA content (PI+) due to preceding apoptotic blebbing. However, from the immunological points of view, cells which stain positive for AnxA5 and PI are those that have lost their membrane integrity, are necrotics and therefore bear higher immunogenic potential. For analysis of cell death and its main forms, 1 x 10^5^ cells were transferred in 400 μl of Ringer’s solution (B. Braun, Melsungen, Germany) containing 0.2 μg AnxA5-FITC and 0.4 μg PI. After 30 min of incubation at 4 °C in the dark, the samples were analyzed by flow cytometry. AnxA5 protein was expressed and produced in 293 human embryonic kidney cells (FreeStyle™ 293 Expression System) and purified (life technologies, GENEART, Regensburg, Germany). Labelling with FITC was performed with the FluoroTag™ FITC Conjugation Kit (Sigma Aldrich, St. Louis, MO, USA) according to the manufacturer’s instructions.

Since the amount of DNA differs between the phases in the cell cycle, a staining of it with PI in the presence of detergent (to allow PI to pass the cell membrane) can be used to analyze the cell cycle phases [47]. Cells in the G2 phase of the cell cycle have a doubled DNA content compared to those in the G1 phase. Degraded DNA (subG1 DNA content of a cell) can also be analyzed by this method, identifying late apoptotic/secondary necrotic cells that already have exported degraded DNA via apoptotic blebs [35]. In brief, cells (5 × 10^5^) were fixed at least for 20 min in 70 % ethanol at −20 °C. After permeabilization of cells with TritonX-100 containing solution, cells were incubated for 30 min with 200 μg/ml RNase A (Roche, Mannheim, Germany) and 5 μg/ml PI at room temperature. An EPICS XL MCL (Beckman Coulter, USA) flow cytometer was utilized for both, cell death and cell cycle analyses.

### Detection of the danger signal Hsp70 and of HMGB1

Heat shock proteins are a set of proteins performing different tasks depending on their location. Inside cells they act as chaperones helping in correct protein-folding, especially preventing dangerous misfolded intermediates being built during cell stress situations. When released from cells, they can act as danger signals such as HMGB1 and are potent stimulators of the adaptive and innate immune system [48]. For detection and quantification of Hsp70 in supernatants of tumor cells the enzyme-linked-immunosorbent assay (ELISA) DuoSet IC Kit (R&D Systems, USA) was used according to the manufacturer’s instructions. HMGB1 was quantified with the ELISA of Shino-Test Corporation (Kanagawa, Japan) also according to the manufacturer’s instructions.

### Differentiation, maturation and activation of human dendritic cells (DCs)

Peripheral blood mononuclear cells (PBMCs) were obtained from human leukocyte cones (ethical approval Nr. 180_13B of IMMO-TuKo-01) using Ficoll-gradient induced blood fragmentation. Therefore the content of a 10 ml leukocyte cone, repeatedly washed with in total 40 ml PBS, was added to four Greiner blue cap centrifuge tubes (Sigma-Aldrich, Germany) containing 12,5 ml Lymphoflot (Bio-rad, Germany) and centrifuged for 20 min at 850 x g. Afterwards the PBMCs, building a proper layer, could easily be separated. The gathered PBMCs were washed three times with 4 °C cold PBS-EDTA (1 ml 0,5 molar PH 7,5 EDTA on 500 ml PBS) and one time with Roswell Park Memorial Institute (RPMI) medium (Lonza, Switzerland) before being seeded out in a concentration of 3,5 x 10^7^ PBMCs per 10 ml RPMI medium refined with 1 % heat inactivated, 0,22 μm filtered human plasma (Sigma, USA), 1 % L-Glutamine (Gibco, United Kingdom) and 0,4 μl/ml Gentamicin (Gibco, United Kingdom) on 100 mm x 20 mm BD Falcon Tissue Culture Dishes (BD Biosciences, USA).

PBMCs were differentiated to DCs by adding 250 U IL4 (PeproTech, Rocky Hill, NJ) and 800 U GM-CSF (PeproTech, Rocky Hill, NJ) per mL medium on day 1, 3 and 5 after isolation according to the protocol of Sallusto et al. [49]. On day 6, the cells were incubated with 10 ml supernatant of approximately 5 x 10^5^ treated tumor cells. 48 h afterwards, the surface expression of different DC activation markers was analyzed using fluorescence labeled antibodies (CD25-FITC (Beckman Coulter, France) , CD40-PerCP/Cy5.5 (Biolegend, USA), CD70-PE (BD Biosciences, USA), CD80-PC7 (Biolegend, USA) , CD83-PE (BD Biosciences, USA), CD86-PerCP/Cy5.5 (Biolegend, USA), DC-SIGN-APC (eBioscience, USA), HLA-DR-Pacific blue (Beckman Coulter, France) and CCR7-PC7 (BD Biosciences, USA)) and a Gallios flow cytometer (Beckman Coulter, USA).

### Statistical analysis

Data were expressed as the mean ± standard deviation (SD) and analyzed for statistical significance using Students t-test (two-tailed) and that for danger signal release by Two-Way ANOVA test. A *p* value <0.05 was considered to be significant and one of <0.01 as highly significant. Comparison of linear-quadratic cell survival curves was performed by an F-Test calculated with the R package ‘CFAssay’ (version 1.2.0.) on R 3.2.1 [45].

## Results

### Irradiation reduces the colony formation of caspase-3 deficient MCF-7 to a greater extent than in caspase-3 proficient MDA-MB231 cells

**Fig. 1:**
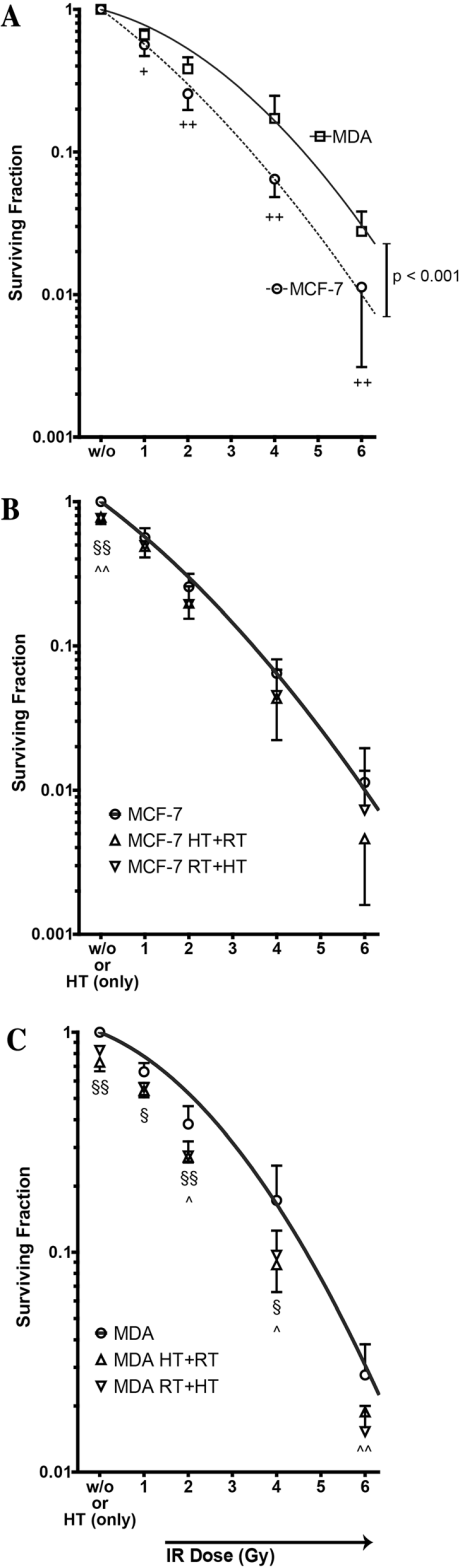
Colony formation of MCF-7 and MDA-MB231 breast cancer cells after irradiation and/or hyperthermia. MCF-7 (**a, b**) or MDA-MB231 (**a, c**) tumor cells were irradiated with ionizing radiation (IR) and/or treated with hyperthermia (41,5 °C for 1 h). The cells were irradiated 4 h before or after HT treatment. The dose-dependent clonogenic potential (surviving fraction) was determined after 2 weeks of incubation. Representative data of one out of three experiments, each performed in triplicates, are presented as mean ± SD. Gy: Gray; HT: hyperthermia; w/o: mock treated control. + *p* <0,05, ++ *p* <0,01; § *p* <0,05, §§*p* <0,01 HT + RT compared to RT only treated; ^ *p* <0,05, ^^ *p* <0,01 RT + HT compared to RT only treated

MDA-MB231 cells are more resistant to radiation as MCF-7 cells when regarding the colony formation capability (Fig. 1a). However, MDA-MB231 cells respond slightly better to addition of HT to irradiation than MCF-7 cells (Fig. 1b), since the surviving fraction was significantly further reduced when adding HT before or after irradiation (RT) (Fig. 1c).

### MCF-7 cells are resistant to cell death induction by irradiation

**Fig. 2:**
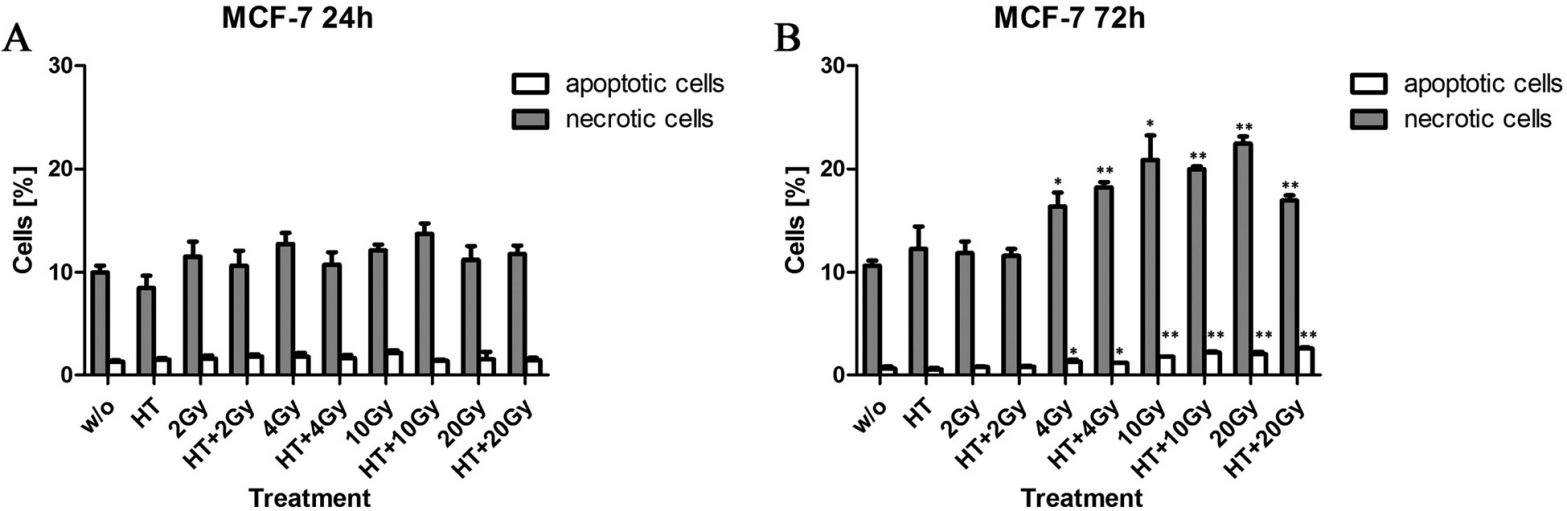
Forms of MCF-7 breast cancer cell death after irradiation and/or hyperthermia. MCF-7 breast cancer cells were irradiated with single doses of ionizing radiation and/or treated with hyperthermia (41,5 °C for 1 h). The cells were irradiated 4 h after HT treatment. 24 h (**a**) or 72 h (**b**) after the last treatment, the cells were stained with AnxA5-FITC/PI and cell death was analyzed by flow cytometry. The percentages of apoptotic (AnxA5+/PI-) and necrotic (AnxA5+/PI+ and PI++) tumor cells are displayed. Representative data of one out of two experiments, each performed in triplicates, are presented as mean ± SD. Gy: Gray; HT: hyperthermia; w/o: mock treated control. *p <0,05, **p <0,01 related to w/o

In contrast to the reduced clonogenicity of MCF-7 cells after RT, but corresponding to deficiency of caspase-3, MCF-7 cells are resistant to cell death induction by RT, HT and combined treatments (Fig. 2a). Not earlier than 72 h after treatment, only a slight increase of apoptotic and necrotic MCF-7 cells was observed, starting at an irradiation dose of 4Gy (Fig. 2b). Of note is that HT applied before (Fig. 2) or after (**not shown**) RT did not impact on the percentage of apoptotic and necrotic MCF-7 cells.

### Radiation induces a mixture of apoptotic and necrotic MDA-MB231 cells

**Fig. 3:**
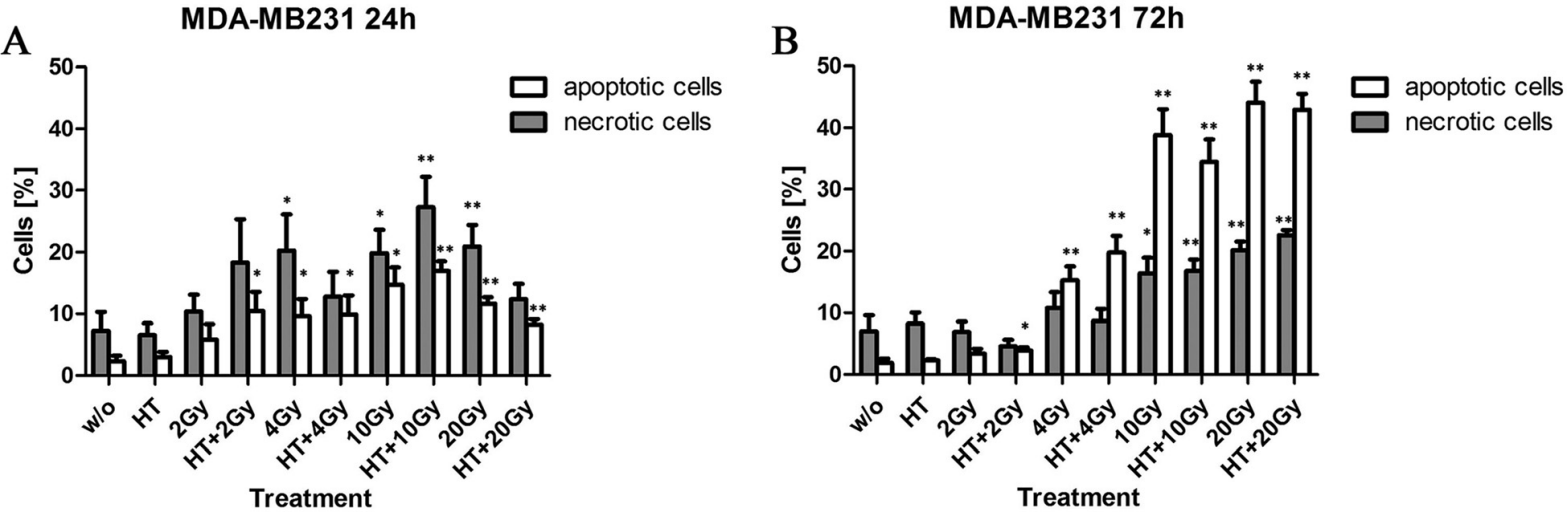
Forms of MDA-MB231 breast cancer cell death after irradiation and/or hyperthermia. MDA-MB231 breast cancer cells were irradiated with single doses of ionizing radiation and/or treated with hyperthermia (41,5 °C for 1 h). The cells were irradiated 4 h after HT treatment. 24 h (**a**) or 72 h (**b**) after the last treatment, the cells were stained with AnxA5-FITC/PI and cell death was analyzed by flow cytometry. The percentages of apoptotic (AnxA5+/PI-) and necrotic (AnxA5+/PI+ and PI++) tumor cells are displayed. Representative data of one out of two experiments, each performed in triplicates, are presented as mean ± SD. Gy: Gray; HT: hyperthermia; w/o: mock treated control. *p <0,05, **p <0,01 related to w/o

In contrast to MCF-7 cells, caspase-3 proficient MDA-MB231 (Fig. 3) cells underwent apoptotic and necrotic cell death already 24 h after irradiation with 4Gy or higher doses (Fig. 3a). A mixture of apoptotic and necrotic tumor cells was induced, while necrosis slightly dominated over apoptosis. HT did not significantly impact on cell death induction of MDA-MB231 by RT. Three days after the treatments, apoptotic MDA-MB231 cells dominated over necrotic ones and a high percentage of apoptotic cells was induced by RT, again starting at a single radiation dose of 4Gy (Fig. 3b).

Hence especially hypofractionated RT should be promising with regard to cell death induction of breast cancer cells. We therefore focused in the subsequent experiments on 4x4Gy vs. 6x3Gy fractionation schemes which have been also proposed for RT treatment of recurrent breast cancer in combination with HT by Oldenborg et al. [23].

### Hypofractionated RT induces apoptosis and necrosis of MDA-MB231 cells

**Fig. 4:**
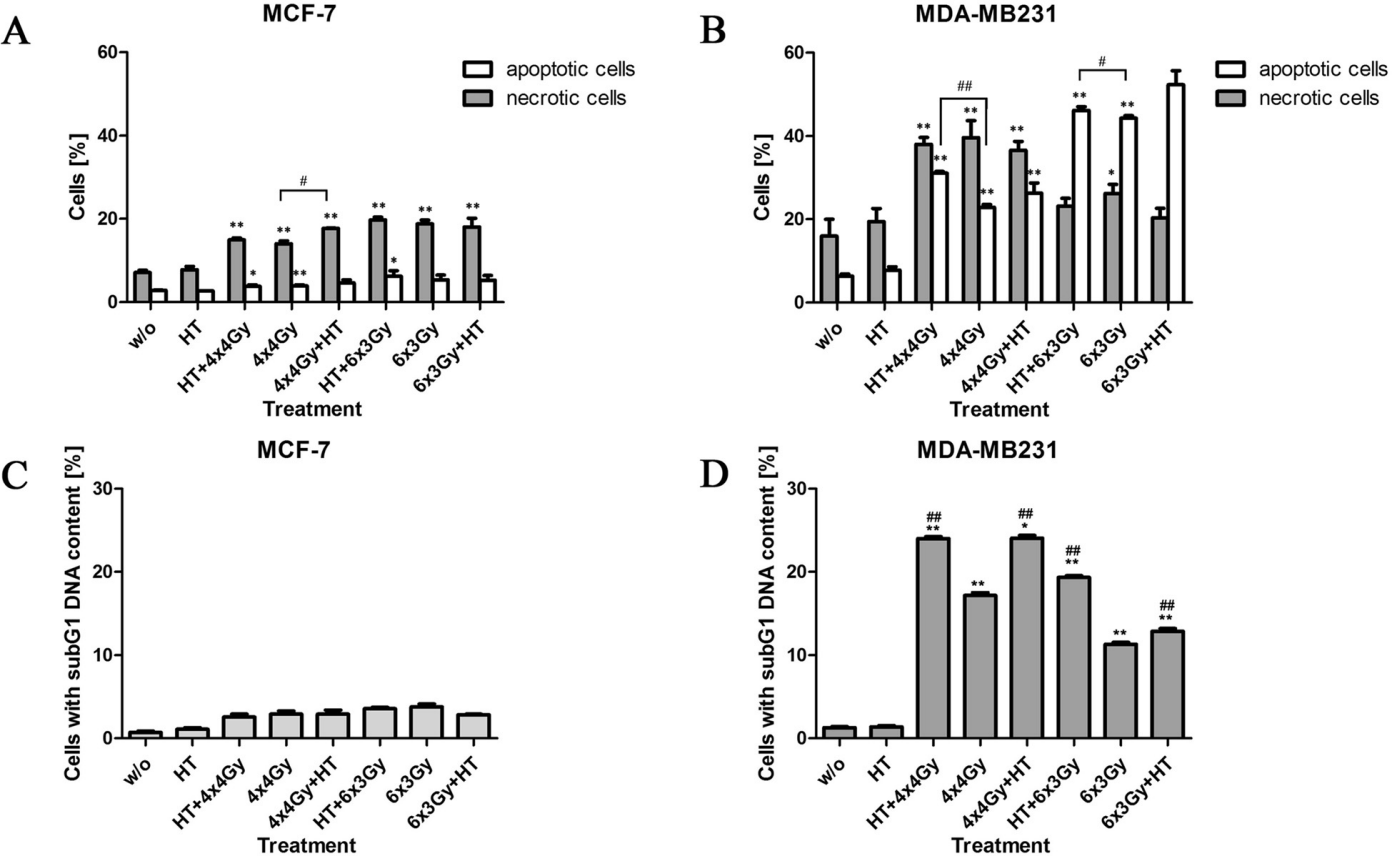
Forms of MCF-7 and MDA-MB231 breast cancer cell death after hypofractionated irradiation and/or hyperthermia. MCF-7 (**a, c**) or MDA-MB231 (**b, d**) breast cancer cells were treated with hypofractionated irradiation (4x4Gy or 6x3Gy) and/or hyperthermia (41,5 °C for 1 h). Ionizing irradiation was applied 4 h before or after hyperthermia on the first and last day of fractionation. 24 h after the last treatment, the cells were harvested. The cells were then stained with AnxA5-FITC/PI and cell death was analyzed by flow cytometry. The percentages of apoptotic (AnxA5+/PI-) and necrotic (AnxA5+/PI+ and PI++) tumor cells are displayed in (**a** and **b)**. For determination of the DNA content, the cells were stained with PI in the presence of detergent and also analyzed by flow cytometry. The percentage of cells with subG1 DNA-content is displayed in (**c** and **d)**. Representative data of one out of three experiments, each performed in triplicates, are presented as mean ± SD. Gy: Gray; HT: hyperthermia; w/o: mock treated control. *p <0,05, **p <0,01 compared to w/o; #*p* <0,05, ##*p* <0,01 related to only irradiated samples

As already observed for irradiation with a single dose, MDA-MB231 cells underwent apoptosis and necrosis also after hypofractionated irradiation with 4x4Gy or 6x3Gy, already 24 h after the last treatment (Fig. 4b). Adding HT to RT resulted in a slight increase of apoptotic MDA-MB231 cells. The latter are most likely late apoptotic ones with still intact membrane integrity, since a significant increase of MDA-MB231 with subG1 DNA content was also observed when adding HT to hypofractionated RT (Fig. 4d). Hypofractionated RT in general induced a high percentage of MDA-MB231 cells with subG1 DNA content (Fig. 4d), while only few MCF-7 cells (<5 %) had already degraded DNA (Fig. 4c). In general, fractionated RT induced only low percentages of necrotic and apoptotic MCF-7 cells (Fig. 4a).

### Hypofractionated RT especially in combination with HT induces the release of Hsp70 by MDA-MB231, but not by MCF-7 cells

**Fig. 5:**
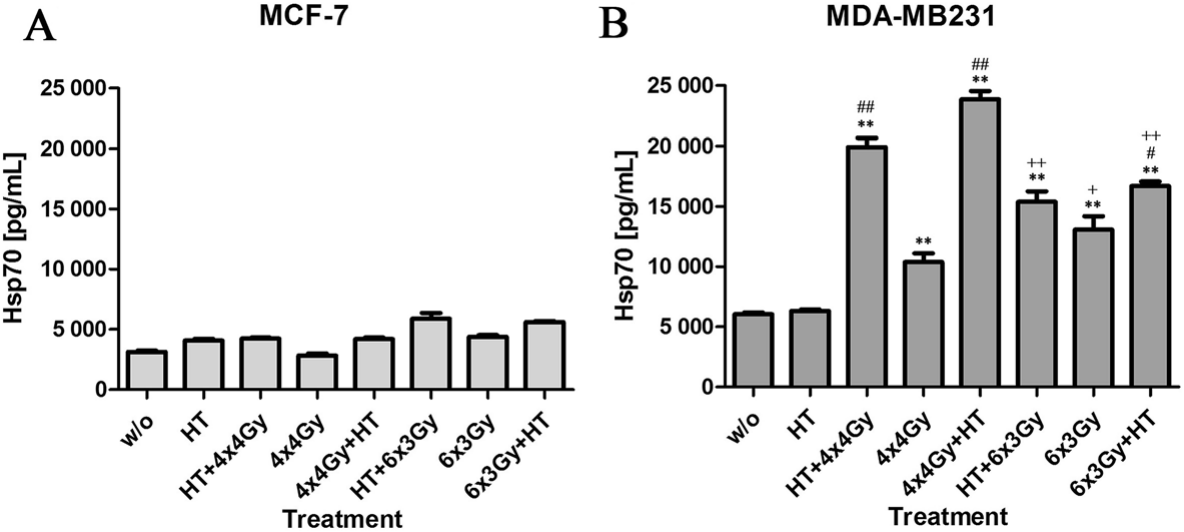
Hsp70 release of MCF-7 and MDA-MB231 breast cancer cells after hypofractionated irradiation and/or hyperthermia. MCF-7 (**a**) or MDA-MB231 (**b**) breast cancer cells were treated with hypofractionated irradiation (4x4Gy or 6x3Gy) and/or hyperthermia (41,5 °C for 1 h). Ionizing irradiation was applied 4 h before or after hyperthermia on the first and last day of fractionation. 24 h after the last treatment, supernatants were analyzed for the amount of extracellular heat shock protein 70. Representative data of one out of four experiments, each performed at least in triplicates, are presented as mean ± SD. Gy: Gray; HT: hyperthermia; Hsp70: heat shock protein 70; w/o: mock treated control. *p <0,05, **p <0,01 compared to w/o; #*p* <0,05, ##*p* <0,01 compared to irradiated samples; +*p* <0,05, ++*p* <0,01 comparing 4x4Gy vs 6x3Gy treatments

Concomitant with the low cell death induction by fractionated RT and/or HT in MCF-7 cells, no significant increased concentrations of the danger signal Hsp70 were observed in the supernatants of the tumor cells after the respective treatments (Fig. 5a). However, again in MDA-MB231 cells, along with the high percentage of cells with subG1 DNA content (Fig. 4d), especially hypofractionated RT in combination with HT induced the release of Hsp70 in the tumor cells supernatant (Fig. 5b).

### Radiation-induced apoptosis and secondary necrosis of MDA-MB231 cells is dependent on caspase-3

**Fig. 6:**
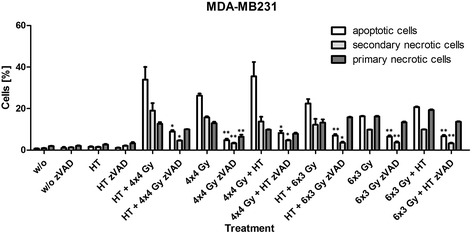
Impact of caspase inhibition on forms of MDA-MB231 breast cancer cell death after hypofractionated irradiation and/or hyperthermia. MDA-MB231 breast cancer cells were treated with hypofractionated irradiation (4x4Gy or 6x3Gy) and/or hyperthermia (41,5 °C for 1 h). The pan-caspase inhibitor zVAD-fmk was added to the cell cultures in a concentration of 50 μM at day one and three or four during hypofractionated irradiation with 4x4Gy or 6x3Gy, respectively. Ionizing irradiation was applied 4 h before or after hyperthermia on the first and last day of fractionation. 24 h after the last treatment, the cells were harvested. The cells were then stained with AnxA5-FITC/PI and cell death was analyzed by flow cytometry. The percentages of apoptotic (AnxA5+/PI-), secondary necrotic (AnxA5+/PI+) and primary necrotic (AnxA5+/PI++) tumor cells are displayed. Representative data of one out of two experiments, each performed in triplicates, are presented as mean ± SD. Gy: Gray; HT: hyperthermia; w/o: mock treated control; zVAD: pan caspase inhibitor zVAD-fmk. *p <0,05, **p <0,01 compared to samples without zVAD

To test whether the deficiency of caspase-3 in MCF-7 cells is the main reason for the lower response regarding cell death induction compared to MDA-MB231 cells, the pan-caspase inhibitor zVAD-fmk was added to MDA-MB231 cells twice during hypofractionated irradiation. As shown in Fig. 6, the addition of zVAD-fmk resulted in significant reduced percentages of apoptotic and secondary necrotic MDA-MB231 cells under all irradiation conditions.

### MDA-MB231 release low amounts of HMGB1 after hypofractionated RT which is dependent on caspase-3 as the Hsp70 release

**Fig. 7:**
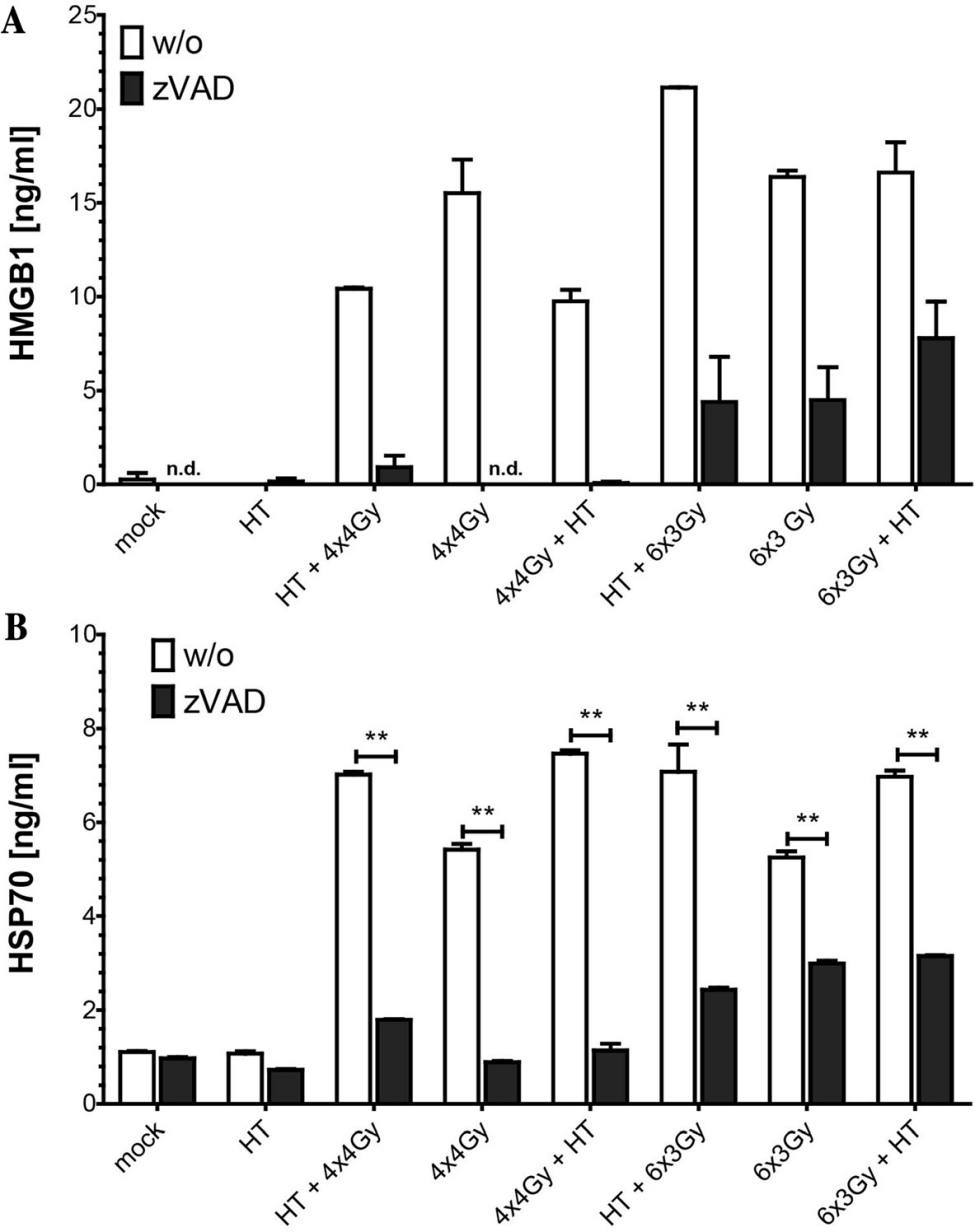
Impact of caspase inhibition on HMGB1 and Hsp70 release by MDA-MB231 breast cancer cells after hypofractionated irradiation and/or hyperthermia. MDA-MB231 breast cancer cells were treated with hypofractionated irradiation (4x4Gy or 6x3Gy) and/or hyperthermia (41,5 °C for 1 h). Ionizing irradiation was applied 4 h before or after hyperthermia on the first and last day of fractionation. The pan-caspase inhibitor zVAD-fmk was added to the cell cultures in a concentration of 50 μM at day one and three or four during hypofractionated irradiation with 4x4Gy or 6x3Gy, respectively. 24 h after the last treatment, supernatants were analyzed for the amount of extracellular HMGB1 (**a**) and Hsp 70 (**b**). Representative data of one out of two experiments, each performed at least in triplicates, are presented as mean ± SD. Gy: Gray; HT: hyperthermia; HMGB1: high mobility group box protein 1; Hsp70: heat shock protein 70; n.d.: not detectable; w/o: mock treated control; zVAD: pan caspase inhibitor zVAD-fmk. **p <0,01 compared to samples without zVAD

While the danger signal HMGB1 was not detected in supernatants of MCF-7 cells (not shown), low concentrations (below 20 ng/ml) were detectable after hypofractionated irradiation (Fig. 7a). Blocking apoptosis and consecutive secondary necrosis in MDA-MB231 by zVAD-fmk decreased HMGB1 in the supernatants of the tumor cells. The same was observed for Hsp70, where addition of zVAD-fmk to the cell cultures significantly reduced the radiation- and radiation plus HT-induced Hsp70 release (Fig. 7b).

### Hypofractionated RT of MDA-MB231 but not of MCF-7 cells creates tumor cell supernatants that slightly increase the expression of activation markers on dendritic cells

**Fig. 8:**
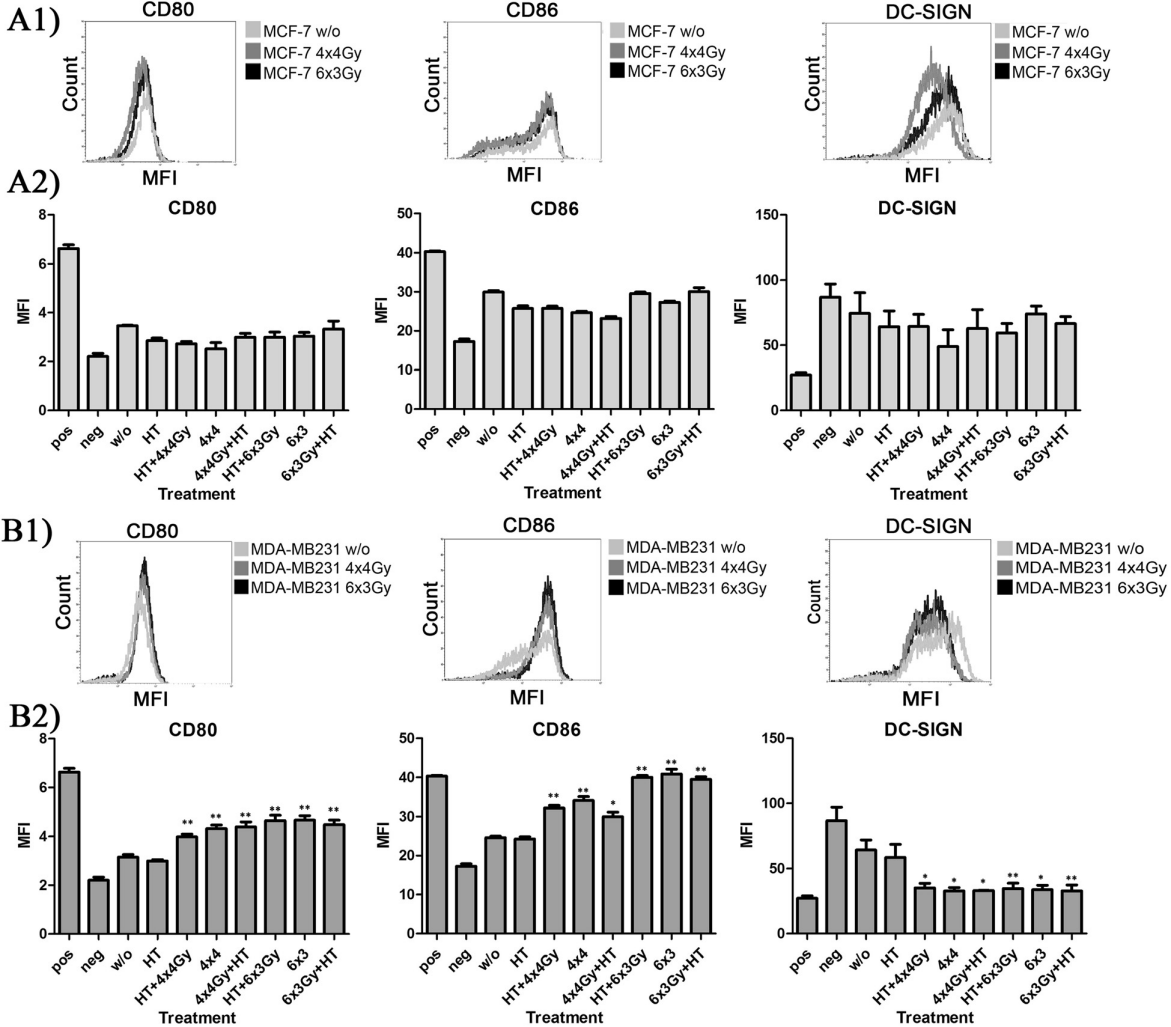
Surface expression of activation markers by dendritic cells after contact with supernatants of MCF-7 or MDA-MB231 breast cancer cells. The surface expression of CD80, CD86, and DC-SIGN on DCs was analyzed by flow cytometry (representative primary data are displayed in **a1** and **b1**) after incubation for two days of immature DCs with cell culture supernatants of MCF-7 (**a**) or MDA-MB231 (**b**) tumor cells treated with hypofractionated irradiation (4x4Gy or 6x3Gy) and/or hyperthermia (41,5 °C for 1 h). Ionizing irradiation was applied 4 h before or after hyperthermia on the first and last day of fractionation. Representative data of one out of three experiments, each performed in triplicates, are presented as mean ± SD in **a2** for MCF-7 cells and **b2** for MDA-MB231 cells. DCs: dendritic cells; Gy: Gray; HT: hyperthermia; MFI: mean fluorescence intensity; w/o: mock treated control; neg: immature DCs in medium only; pos: immature DCs incubated with IL-1β, PGE2, IL-6 and TNFα instead of tumor cell supernatants; *p <0,05, **p <0,01 compared to w/o

After co-incubation for two days of human monocyte-derived DCs with supernatants (SNs) of MCF-7 cells, no significant changes in the surface expression of activation markers on DCs could be observed when comparing SNs of untreated (w/o) or RT and/or HT treated cells (Fig. 8a1 and a2). Of note is that the untreated MCF-7 cells are per se more immunogenic compared to MDA-MB231 cells (Fig. 8). However, SNs of irradiated MDA-MB231 cells with lower basal immunogenicity induced a significant increased surface expression of the activation markers CD80 and especially of CD86 on DCs (Fig. 8b1 and b2). Concomitantly, the surface expression of DC-SIGN was significantly reduced, also a clue for the activation of DCs. A slight, but not significant increase of the DC homing receptor CCR7 which was generally very low expressed was also seen after incubation of DCs with supernatants of MDA-MB231 cells that had been treated with hypofractionated RT (not shown). Of note is that HT alone had no effects on the expression of activation markers on DCs (Fig. 8).

## Discussion

Although the last decades brought a wide enlargement of breast cancer therapies, there are still several limitations which need to be vanquished [50]. Advances in chemo- and radiotherapy, surgical techniques and the development of new targeted agents have significantly improved clinical outcomes for patients with locally advanced breast cancer [51]. Radiotherapy is an integral part of the therapy for local tumor control and we observed that the triple negative but caspase 3 proficient MDA-MB231 cells are less radiosensitive than the MCF7 cells regarding the clonogenicity (Fig. 1). However, higher amounts of apoptotic and necrotic cells are induced by irradiation starting at 4Gy in MDA-MB231 cells compared to MCF-7 cells (Fig. 2). This highlights that definition of “radiosensitivity” is diverse in dependence of the focus on tumor cells clonogenicity or cell death, respectively, and that cell death is dispensable for radiation-induced reduction of clonogenicity [27, 52]. Along the same line, while HT did not impact on cell death induction (Fig. 3), it slightly, but significantly reduced the surviving fraction based on the clonogenicity of MDA-MB231 cells (Fig. 1c). Further, addition of HT to hypofractionated RT reduced the amount of cells in the radiosensitive G2 cell cycle phase only in MDA-MB231 slightly, but significantly (Additional file 1: Figures S1). This could indicate that p53 mutated tumor cells such as MDA-MB231 response to HT by reduction of radiation-induced G2 arrest, as also observed for checkpoint kinase inhibitors [53].

While for the aggressive triple-negative breast cancer subtype innovative treatment methods based mainly on chemotherapy, biologic agents and radiotherapy show also good tumor control, a limited time of disease free survival is still the reality [54]. This implies that especially systemic immune activation might be beneficial for fighting metastases and recurrent tumors [38]. For activation of the immune system the endoplasmatic reticulum (ER) stress and forms of tumor cell death are decisive and not the clonogenicity of the tumor cells themselves [55, 56]. Hypofractionated irradiation was used in our preclinical *in vitro* assays, since 10 years follow-up confirmed that hypofractionated RT is safe and effective for patients with early breast cancer [10]. In preclinical model systems, abscopal and immune mediated effects in breast cancer are mainly observed when combining hypofractionated irradiation with further immune stimulation [57]. We focused on hyperthermia as a promising enhancer of RT concerning direct tumor cell cytotoxicity, but also as a stimulator of the immune system by generating an *in situ* tumor vaccine [17]. Here, DCs are central since they link innate and adaptive immune responses and are stimulators of CD8+ cytotoxic T cell responses. Such tumor infiltrating lymphocytes (TILs) have been demonstrated to predict higher pathologic complete response rates in triple negative patients [58, 59].

Our data suggest that caspase-3 proficient MDA-MB231 can be rendered more immunogenic by hypofractionated irradiation. Treatment of these cells with ionizing radiation resulted in a mixture of apoptotic and necrotic cells at doses of 4Gy and above (Fig. 3a and b). This mixture of dying and dead tumor cells is supposed to bear a high immunogenic potential [60]. When MDA-MB231 cells were treated with 4x4Gy or 6x3Gy hypofractionated irradiation, also a mixture of apoptotic and necrotic cells resulted (Figs. 4 and 6). HT did impact on the percentage of cells with subG1 content (Fig. 4d) and the release of the danger signal Hsp70 (Figs. 5b and 7b): both were increased when HT was added to hypofractionated RT in MDA-MB231 cells.

In MCF-7 cells, cell death induction by RT was much weaker and HT did not increase the amount of late apoptotic cells with increased subG1 DNA content nor resulted in release of Hsp70 or HMGB1. This could suggest that especially combination of hypofractionated RT with HT in caspase-3 proficient cells such as MDA-MB231 cells generates tumor cell SNs that lead to increased expression of activation markers on DCs. However, the expression of the CD80 and CD86 on DCs was increased when incubating immature DCs with SNs of MDA-MB231 cells that had been exposed to hypofractionated irradiation. Addition of HT did not significantly impact on it (Fig. 8b). Concomitantly, a decreased expression of DC-SIGN was detected. Since ligation of DC-SIGN on DCs actively primes DCs to induce Tregs, a reduced expression is favorable for induction of anti-tumor immune responses [61]. Regarding the homing receptor CCR7 on DCs, also a slight, but not significant, increased expression was observed after hypofractionated RT (not shown). This is favorable for directing the DCs to the next lymph nodes where they then prime naïve T cells [62]. We have previously shown that the SNs of colorectal tumor cells that had been treated with RT plus HT also induce a significant up-regulation of expression of CD80 and CCR7 on DCs and that Hsp70 is one mediator of it. However, Hsp70 is not the sole stimulus for this, since HT treatment alone resulted in a comparable release of Hsp70 compared to RT plus HT, while only a very slight increase of expression of CD 80 and CCR-7 on DCs was observed in the case of only HT application [44]. In the case of the MDA-MB231 breast cancer cells, slightly higher concentrations of Hsp70 in tumor cell SNs after combination of fractionated RT with HT is not the key stimulus for increased surface expression of activation markers on DCs (Fig. 8). This necessitates that further immune stimulatory molecules besides Hsp70s, which have to be identified in future work, are released by the breast cancer cells. HMGB1 might be one component of it, since its concentration is slightly increased in MDA-B231 cells, but not in MCF-7 cells, only in dependence of irradiation (Fig. 7a). The increased amount of Hsp70 after combination of hypofractionated RT with HT might rather lead to activation of NK cells than to further stimulate DCs [63, 64].

Caspases are central for most apoptosis inductions [65]. We proved that apoptosis and consecutive secondary necrosis-induction by hypofractionated irradiation of breast cancer cells is dependent on caspase-3 and can therefore be efficiently blocked by the pan-caspase inhibitor zVAD-fmk (Fig. 6). We conclude that hypofractionated RT induces cell death only in caspase-3-proficient breast cancer cells. Addition of HT to hypofractionated RT fosters the release of Hsp70 by these cells, but does not generate tumor cell SNs that do further increase the hypofractionated radiation-dependent enhanced expression of activation markers on DCs (Fig. 8).

We conclude that, in the light of personalized medicine, for the assessment of radiosensitivity and local as well as expected systemic cancer treatment efficacy of distinct fractionation schemes of radiation and additional immunotherapies for breast cancer, the readout system is crucial and has to be announced stringently. As demonstrated, clonogenicity is not primarily linked to caspases: caspase deficient MCF-7 cells are reduced in their clonogenic potential more than MDA-B231 cells. However, an additional stimulus such as HT might differentially impact on it, since only in MDA-B231 cells the clonogenic potential was further reduced by adding HT to irradiation. Cell death induction and distinct forms of cell death on the contrary reflect the killing efficacy of treatments and is connected to caspase integrity: Higher amounts of apoptotic and necrotic cells are induced in MDA-B231 cells by especially irradiation. To gain first hints about the immunogenic potential of the cancer cells, analyses of released danger signals should be performed. MDA-B231 cells released higher amounts of Hsp70 and HMGB1 after fractionated RT. However, only the release of Hsp70 was further increased by HT. Further, continued testing as incubation of the SNs with DCs is necessary. Our data revealed that HT did not further increase the hypofractionated radiation-dependent enhanced expression of activation markers on DCs. Finally, upregulation of activation markers on DC might also result in tolerance, so *in vivo* testing with syngenic mouse models should be increasingly performed in the future.

Nevertheless, the multiple *in vitro* assays that we used for our study allow to draw the conclusion that hypofractionated irradiation is one main stimulus for cell death induction and consecutive DC activation in caspase-3 proficient breast cancer cells. Hennel et al. demonstrated that ablative RT, also as single application, potently induces necrosis in fast proliferating, hormone receptor negative breast cancer cell lines with mutant p53 [66]. This in turn stimulates monocyte migration and most likely consecutive immune activation. Therefore, both, radiation with higher single doses applied in ablative and hypofractionated RT seem to be a key stimulus for DC activation by SNs of caspase proficient tumor cells.

Additional immune modulation with HT fosters the release of higher amounts of Hsp70 and therefore might result in activation of NK cells. Since synergistic effects of innate and adaptive immunity are beneficial for the induction of anti-tumor immunity and especially for immunological memory [67] it should also be the aim to activate both CD8+ T cells via DCs and NK cells for therapy of selected breast cancer types. Besides consideration of disease extent, host factors, patient preferences and social and economic constraints [68], the configuration of tumor cells with cell death executing proteins such as caspases should be included in radio-immunotherapy decisions to enhance the immunogenicity of distinct breast cancers by hypofractionated RT alone or in combination with further immune modulation in a personalized manner.

## Additional file

Additional file 1: Figure S1. MCF-7 and MDA-MB231 breast cancer cells in the G2 cell cycle phase after hypofractionated irradiation and/or hyperthermia. MCF-7 (A) or MDA-MB231 (B) tumor cells were treated with hypofractionated irradiation (4x4Gy or 6x3Gy) and/or hyperthermia (41,5 °C for 1 h). Ionizing irradiation was applied 4 h before or after hyperthermia on the first and last day of fractionation. 24 h after the last treatment, the cells were harvested. For determination of the DNA content, the cells were stained with PI in the presence of detergent and analyzed by flow cytometry. The percentage of cells in the G2-phase of the cell cycle is displayed. Representative data of one out of three experiments, each performed in triplicates, are presented as mean ± SD. Gy: Gray; HT: hyperthermia; w/o: mock treated control. **p* <0,05, ***p* <0,01 compared to w/o; # *p* <0,05, ## *p* <0,01 compared to only irradiated samples. (JPEG 359 kb)
