# Supplementary figures and images for: The in vitro immunogenic potential of caspase-3 proficient breast cancer cells with basal low immunogenicity is increased by hypofractionated irradiation
Source: Radiat Oncol. 2015 Sep 17;10:197. doi: 10.1186/s13014-015-0506-5 (PMC4573696; doi:10.1186/s13014-015-0506-5)

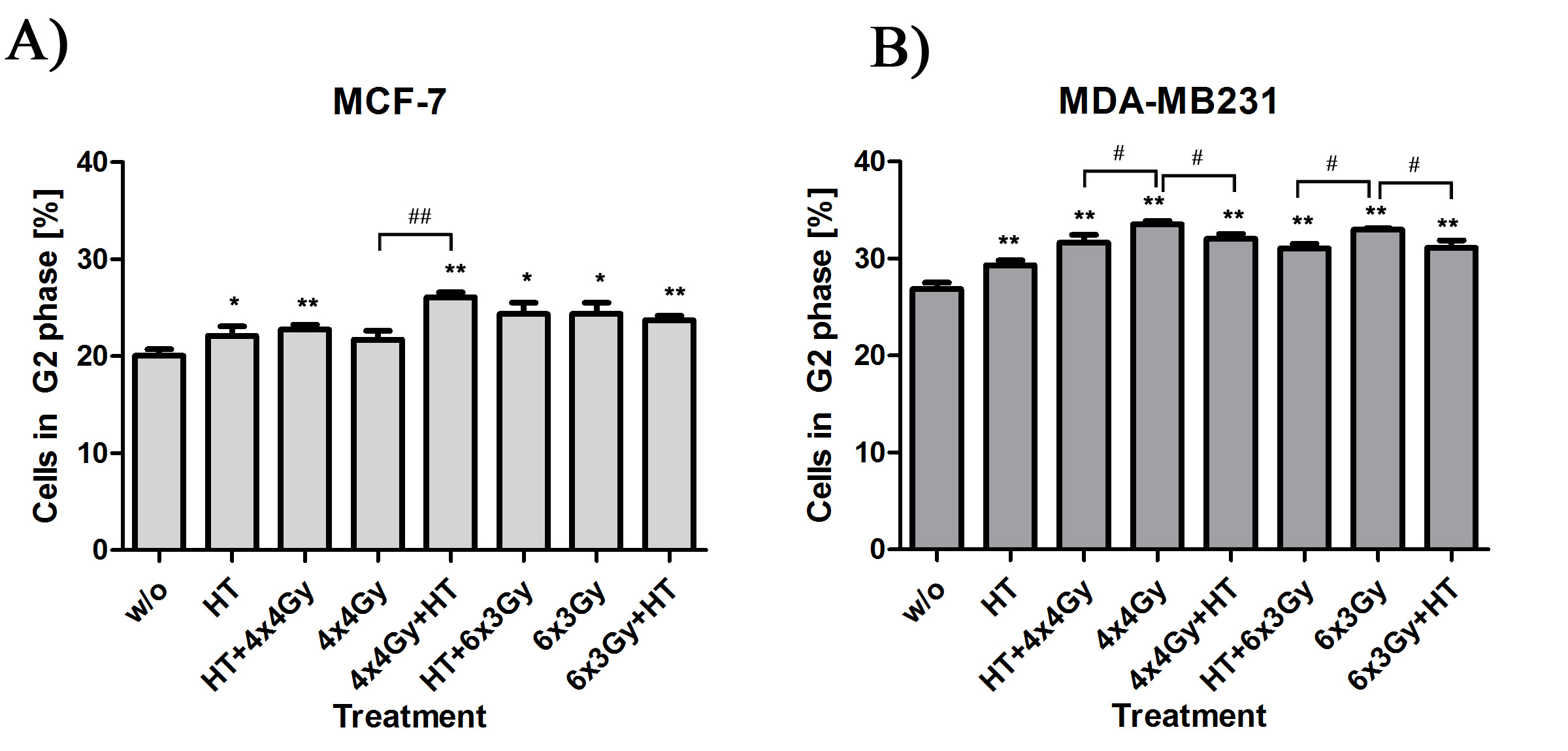

Supplement: Additional file 1: Figure S1. — MCF-7 and MDA-MB231 breast cancer cells in the G2 cell cycle phase after hypofractionated irradiation and/or hyperthermia. MCF-7 (A) or MDA-MB231 (B) tumor cells were treated with hypofractionated irradiation (4x4Gy or 6x3Gy) and/or hyperthermia (41,5 °C for 1 h). Ionizing irradiation was applied 4 h before or after hyperthermia on the first and last day of fractionation. 24 h after the last treatment, the cells were harvested. For determination of the DNA content, the cells were stained with PI in the presence of detergent and analyzed by flow cytometry. The percentage of cells in the G2-phase of the cell cycle is displayed. Representative data of one out of three experiments, each performed in triplicates, are presented as mean ± SD. Gy: Gray; HT: hyperthermia; w/o: mock treated control. *p <0,05, **p <0,01 compared to w/o; # p <0,05, ## p <0,01 compared to only irradiated samples. (JPEG 359 kb) [file 13014_2015_506_MOESM1_ESM.jpg › ESM/13014_2015_506_MOESM1_ESM.jpg]
